# Not surprising: a rebound in antibacterial consumption in Europe, with Cyprus and Greece on the podium

**DOI:** 10.1093/jac/dkae055

**Published:** 2024-03-05

**Authors:** Nikolaos Spernovasilis, Constantinos Tsioutis

**Affiliations:** Department of Infectious Diseases, German Oncology Centre, 4108 Limassol, Cyprus; School of Medicine, University of Crete, 71500 Heraklion, Greece; School of Medicine, European University Cyprus, 6 Diogenes str, 2404 Nicosia, Cyprus

## Abstract

Recent European-wide data place Cyprus and Greece in the highest positions of total antimicrobial consumption. While this level of consumption might be partly attributed to the high rates of infections due to MDR bacteria in these countries, several other reasons should be sought to help apply local measures, to decrease inappropriate and excess antimicrobial use. The present viewpoint aims to provide a roadmap for interventions, by briefly discussing the various factors that underlie antimicrobial use and prescribing practices in Greece and Cyprus.

Recently, the ECDC reported the updated data on antimicrobial consumption in 29 countries for 2022, comprising 27 EU Member-States and two European Economic Area (EEA) countries, namely Iceland and Norway.^1^ The combined consumption of antibacterials for systemic use in the community and hospital sectors for these countries was significantly increased in 2022 compared with 2020 and 2021, with a population-weighted mean consumption of 19.4 DDD per 1000 inhabitants per day. Even though all European countries marked an increase in consumption, Cyprus and Greece ranked first with 33.5 and 32.9 DDD per 1000 inhabitants per day, respectively.^1^ Not unrelatedly, these two countries also have a high rate of infections due to MDR bacteria; for example, in 2022 Cyprus reported the highest percentage of invasive MRSA isolates and Greece reported the highest percentage of invasive *Klebsiella pneumoniae* isolates resistant to carbapenems among all EU/EEA countries,^2^ whereas specifically Greece has been known to be highly prevalent in carbapenem-resistant Gram-negative bacteria for many years now.^3^

During the years 2020 and 2021, these two countries, as most European countries, reported lower consumption of antibacterials for systemic use, not only compared with 2022 but also compared with 2019.^1^ As in all countries with lower consumption during this period, this observation is most probably due to a combination of reasons related to the COVID-19 pandemic, such as lockdowns and improved personal and environmental hygiene, which prevented transmission of infectious diseases other than SARS-CoV-2, the predomination of COVID-19 in the community, which after the initial phases of the pandemic was treated based on specific treatment protocols precluding antibiotic use, and the difficulties related to the access to primary care service, which may have led to a decrease in the overdiagnosis or misdiagnosis of bacterial infections.^4–7^ Nevertheless, this decreased consumption was expected to be transient, as the clinical epidemiology of infectious diseases and associated treatments would inevitably follow the course of the measures that were in effect during the pandemic, thus leading to a new increase in infection rates of various causes in the community and healthcare setting.^8^

Taking into account local evidence, in combination with our 15 year-long background in antimicrobial stewardship and infection prevention and control (IPC) in both Greece and Cyprus, we believe that the high rates of antimicrobial consumption and invasive MDR isolates in these countries can be attributed to several factors, some of which may also apply to other countries of the East Mediterranean region, the Balkans, and other areas with findings of a similar extent. Firstly, the lifting of non-pharmaceutical interventions after the first years of the COVID-19 pandemic has led to an increase of other community pathogens, thus necessitating higher antibiotic use both in the primary care and hospital setting. A relevant example is the reported increase of *Streptococcus pneumoniae* infections in Europe during 2022 compared with 2020 and 2021.^2^ Furthermore, local prescribing drivers in relation to patient pressure and pharmaceutical industry marketing, which resurfaced after strict lockdowns and contact limitations were lifted, might have affected antimicrobial prescribing to a certain degree.^9^ In fact, ‘aggressive’ marketing has been acknowledged as an important driver of inappropriate antimicrobial use among junior doctors in Greece.^9^ Also, previous local surveys have shown that despite a high level of awareness regarding the necessity of prudent antimicrobial use among the general population, important knowledge gaps often lead to patients requesting antimicrobials even though they are not indicated (e.g. in cases of viral infections).^10^ In addition, health system and financial disruptions may have impacted the availability of and access to antimicrobials for non-COVID-19 infections during the pandemic, thus contributing to the lower use of antimicrobials observed in 2020 and 2021.^11^ Within healthcare settings, relaxation of IPC measures may have facilitated an increase of healthcare-associated infections and subsequent use of antimicrobials for their treatment. Lack of local and regional guidance for optimal management of infections, tailored to epidemiological and other local data, as well as limited training in antimicrobial stewardship among prescribers, are also important drivers of inappropriate antimicrobial use.^12^ Finally, various local social and cultural factors affect prescribing behaviours and practices in Cyprus and Greece, which share several similarities in this respect.^9,10^

A multidisciplinary national approach to coordinate actions addressing the above factors is imperative in both countries. Firstly, IPC measures in healthcare settings call for enforcement and regular monitoring, through leadership and management support, continuous surveillance, strengthening infection control teams with resources and manpower, and regular feedback and communication.^3^ Similarly, several community measures have proven to be of benefit, such as education and awareness on personal protection and hand hygiene, avoiding contacts when symptomatic, and adequate indoor ventilation.^13,14^ Additionally, regulation of antimicrobial prescribing through electronic prescription, adherence to local guidance and continuous surveillance of antimicrobial use have been proven to affect the practices and attitudes of prescribers, while decreasing the effects of other parties on prescription practices such as patients and industry.^8,9^ It should be noted that declaration of funding from the industry is a legal requirement for all doctors in Greece but not in Cyprus. Furthermore, in the past few years, antimicrobials have been mandatorily dispensed in the community only through an electronic prescription platform in Greece and Cyprus. It is also worth noting that Cyprus lacks national recommendations for treatment of the majority of infections, a fact which has undoubtedly contributed to inappropriate antimicrobial use in the country, and which should be addressed as soon as possible. Another point of interest is the need for requirement for continuous medical education among doctors, which will contribute to ensuring awareness and education on appropriate prescribing practices.^9^

In conclusion, current evidence suggests that in Cyprus, Greece and other European countries experiencing similar phenomena in antimicrobial consumption rates, coordinated and locally tailored multidisciplinary efforts are urgently needed to address several medical, financial, and sociocultural factors, especially in light of the reported EU target to reduce overall antibiotic consumption by 20% by 2030.^15^
